# An Encapsulated Organic Acid and Essential Oil Mixture Improves the Intestinal Health of Weaned Piglets by Altering Intestinal Inflammation and Antioxidative Capacity

**DOI:** 10.3390/ani12182426

**Published:** 2022-09-15

**Authors:** Aidong Liu, Zhen Li, Xu Jin, Qiong Wu, Hong Hu, Cheng Zhang

**Affiliations:** 1 Department of Animal Science, College of Animal Science and Technology, Anhui Agricultural University, Hefei 230036, China; 2 Department of Animal Science, College of Animal Science, Anhui Science and Technology University, Fengyang 233100, China

**Keywords:** antioxidant, essential oils, inflammation, intestinal health, organic acid, signaling pathway

## Abstract

**Simple Summary:**

Weaning stress causes retarded growth, gut disorder and dysfunction, severe diarrhea and higher mortality in weaned piglets. Antibiotic growth promoters (AGP) have been conventionally used to alleviate the negative effects of weaning stress. However, the long-term use of AGP leads to various adverse effects, such as antimicrobial resistance and food drug residues, and threatens public safety. Therefore, AGP should be replaced with residue-free, pollution-free and toxin-free alternatives because several countries and regions have banned the use of AGP in the feed industry. This study investigates the effects of an encapsulated organic acid and essential oil mixture (OAEO) on the growth performance, immuno-antioxidant capacity and intestinal health of weaned piglets. The results reveal that OAEO as an alternative to AGP improved the growth performance, immuno-antioxidant status and intestinal health of weaned piglets partly by activating the Nrf2 signaling pathway and suppressing the TLR4/NF-κB signaling pathway.

**Abstract:**

This study investigates the effects of an encapsulated organic acid and essential oil mixture (OAEO) on the growth performance, immuno-antioxidant capacity and intestinal health of weaned piglets. In total, 120 weaned piglets (23 days of age; 6.96 ± 0.08 kg) were randomly allotted to four treatments (six replicates/group; five piglets/replicate): the control group (CON) was fed the basal diet (BD), the antibiotic growth promoters group (AGP) received the BD with 20 mg/kg colistin sulphate and 10 mg/kg bacitracin zinc, and OAEO1 and OAEO2 were fed the BD with 1000 mg/kg and 2000 mg/kg OAEO, respectively. The trial lasted 21 days and then one piglet per replicate was selected for sample collection. OAEO increased the average daily gain, spleen index, serum interleukin (IL)-10, immunoglobulin (Ig) G and IgA levels; serum superoxide dismutase and glutathione peroxidase (GPX) activities; and jejunal villus height (VH), VH/crypt depth, goblet cell number, and amylase and trypsin activities (*p* < 0.05) compared with CON but reduced the diarrhea rate, serum tumor necrosis factor (TNF)-α, malondialdehyde (MDA), and _D_-lactic acid contents and diamine oxidase (DAO) activity (*p* < 0.05). OAEO also increased the jejunal zonula occludens-1, occludin, claudin-1, mucin-2, nuclear factor erythroid 2-related factor 2 (Nrf2), GPX and IL-10 mRNA levels, GPX activity and IL-10 content (*p* < 0.05) compared with CON but reduced jejunal MDA, IL-1β and TNF-α contents and Toll-like receptor (TLR) 4, nuclear factor (NF)-κB and TNF-α mRNA levels (*p* < 0.05). In addition, AGP increased ADG, serum IgA level and GPX activity, jejunal trypsin activity and IL-10 content and mRNA level (*p* < 0.05) compared with CON but reduced the serum TNF-α content and DAO activity and jejunal NF-κB mRNA level (*p* < 0.05). Overall, OAEO as an alternative to AGP improved the growth performance, immuno-antioxidant status and gut health of weaned piglets partly via activating the Nrf2 signaling pathway and suppressing the TLR4/NF-κB signaling pathway.

## 1. Introduction

Early weaning is crucial to increase the efficiency of pig production and widely practiced in the pig industry. However, early weaning causes severe weaning stress, which is characterized by retarded growth, gut disorder and dysfunction, severe diarrhea, and increased susceptibility to infections and mortality, eventually resulting in considerable economic loss. Therefore, reducing weaning stress is the key to ensure the successful application of early weaning techniques. In the past few decades, subtherapeutic doses of antibiotics added to feed, also known as antibiotic growth promoters (AGP), have been conventionally used to mitigate the negative effects of weaning stress [1,2]. However, the long-term use of AGP can lead to various hazards, including antimicrobial resistance, the presence of drug residues in food and disruption in the immunity and intestinal flora of animals; this can limit the sustainable development of the pig industry and even threaten public safety [3,4]. For this reason, the use of AGP in feed has been prohibited by the United States, the European Union and China. The restriction of the use of antibiotics in livestock production has become a major challenge for the pig industry [5]. Thus, possible eco-friendly, drug-resistance-free and cost-effective AGP alternatives should be identified to alleviate the weaning stress of weaned piglets.

Various functional feed additives have been investigated as AGP alternatives. Among them, organic acids and essential oils have been determined to be extraordinarily effective [6,7]. Formic acid, fumaric acid, citric acid and benzoic acid are the more frequently used organic acids [8]. In addition, organic acid salts, such as calcium formate and potassium diformate, are commonly used as acidifiers [6]. Organic acids alone or in combination with organic and inorganic acid salts are widely reported to have the potential to replace AGP because they can reduce diarrhea, maintain the balance of intestinal flora, improve digestive enzyme activities and nutrient digestibility, and enhance gut morphology and barrier integrity, ultimately improving the growth performance of piglets [8,9,10]. Essential oils are another widely used feed additive with the potential to replace AGP [11]. The most commonly used essential oils include thymol, eugenol, cinnamaldehyde and carvacrol, which are reported to have high immune, antioxidative, antimicrobial and anti-inflammatory properties, and can thus promote the gut health and growth performance of pigs [11,12,13]. However, a single feed additive cannot achieve the purpose of replacing AGP. The most promising replacement for AGP may be a combination of different feed additives that have the potential to replace antibiotics [7]. Interestingly, it was reported that essential oils could increase the permeability of the bacterial cell membrane, resulting in an increase in undissociated organic acids into the cytoplasm of pathogenic bacteria and thus hampering bacteria’s cellular metabolism by reducing cellular pH [14,15]. A previous study also found that organic acids could reduce the gastrointestinal pH of weaned pigs [16] and thus strengthen the antibacterial activity of essential oils [17]. Moreover, recent studies indicated that the combination of organic acids and essential oils synergistically reduced diarrhea and enhanced growth performance and gut health in weaned piglets [1,5,18]. However, additional studies examining more comprehensive indicators are warranted to determine the beneficial effects of the combination of organic acids and essential oils and elucidate their underlying mechanism of action. Based on all the above facts, this study investigates the effects of the combination of organic acids and essential oils as substitutes for AGP on the growth performance, diarrhea, immuno-antioxidant capacity and gut barrier function of weaned piglets. In addition, the mechanism of action underlying the effects of the combination of organic acids and essential oils on gut health was elucidated by focusing on the nuclear factor erythroid 2-related factor 2 (Nrf2) signaling pathway (a key signaling pathway for regulating antioxidant capacity) and Toll-like receptor 4 (TLR4)/nuclear factor-κB (NF-κB) signaling pathways (a key signaling pathway for regulating inflammatory response) because the combination of organic acids and essential oils showed certain immuno-antioxidant properties in piglets [5].

## 2. Materials and Methods

### 2.1. Experimental Design and Diets

The experimental procedures of the study were approved by the Animal Use and Care Committee of Anhui Agricultural University (approval code: AHAUB002). A total of 120 weaned piglets (Duroc × [Landrace × Yorkshire], weaned at day 23) with an initial average body weight of 6.96 ± 0.08 kg were selected and randomly allocated to four treatments with six replicates containing five piglets each: (1) the control group (CON), in which piglets were fed the basal diet (BD); (2) the AGP group (AGP), in which piglets were fed the BD with 10 mg/kg bacitracin zinc and 20 mg/kg colistin sulphate; (3) encapsulated organic acids and essential oils mixture (OAEO) group 1 (OAEO1), in which piglets were fed the BD with 1 g/kg OAEO; (4) OAEO group 2 (OAEO2), in which piglets were fed the BD with 2 g/kg OAEO. The BD was formulated in accordance with requirements for piglets specified by the National Research Council (2012), and its composition and nutrients are listed in Table S1. The main active ingredients of OAEO were thymol (4.5%), carvacrol (4.8%), cinnamaldehyde (4.3%), calcium formate (10.1%) and citric acid (25.1%) encapsulated with hydrogenated vegetable oils. The feeding experiment lasted 21 days from 22 March 2022 to 11 April 2022. All piglets were raised on the experimental farm (located in Luyang District, Heifei, China) of Anhui Agricultural University with a density of 0.8 m^2^ per piglet and allowed to free intake of water and feed. Daily feed intake was recorded in units of repetition. Each piglet was weighed at the beginning and end of the feeding trial after 12 h fasting. The average daily feed intake (ADFI), average daily gain (ADG) and the ratio of feed intake to body weight gain (F/G) were calculated. The diarrhea score (0 = normal feces, 1 = soft feces, 2 = moderately fluid feces, 3 = very watery and frothy diarrhea) was recorded daily by independent experienced evaluators and the diarrhea rate was calculated as described previously [19].

### 2.2. Sample Collection

At the end of the feeding trial, one piglet with the closest to the average body weight per replicate was selected for sample collection. A 6 mL blood sample was collected from the anterior vena cava of the piglets after 12 h fasting and centrifuged at 3000× *g* for 10 min at 4 °C to obtain the serum sample which was collected in 0.5 mL centrifuge tubes and placed at −80 °C for the subsequent analysis. All selected piglets were exsanguinated after electrical stunning. The spleen, thymus and mesenteric lymph nodes were dissected and weighed, and immune organ indices are expressed relative to body weight (g/kg). Approximately 1.5 cm segments of the mid-jejunum were isolated and fixed in 4% paraformaldehyde solution for morphological evaluation. Adjacent jejunum samples (approximately 10 cm) were removed. Then, the fresh intestinal content was immediately collected in 10 mL centrifuge tubes and placed at −80 °C for the further analysis of digestive enzyme activity. The jejunum samples were opened longitudinally and washed in precooled phosphate-buffered saline (PBS). The mucosa sample was gently scraped into 1.5 mL centrifuge tubes by using a sterile glass slide, immediately placed in liquid nitrogen and preserved at −80 °C for further analysis.

### 2.3. Immunity, Antioxidative Capacity and Enteric Permeability Marker Measurement

The jejunal mucosa was homogenized together with precooled PBS by using a homogenizer. Then, the homogenate was centrifuged at 4000× *g* for 12 min at 4 °C to obtain the supernatant for the analysis of the antioxidative capacity and cytokines contents. The protein concentration of the supernatant was quantitated using a commercially available kit (Nanjing Jiancheng Bioengineering Institute, Nanjing, China). The levels of _D_-lactic acid, immunoglobulin (Ig) G, IgA and IgM in the serum samples and those of interleukin (IL)-1β, IL-6, IL-8, IL-10 and tumor necrosis factor (TNF)-α in the serum and jejunal mucosa samples were measured using enzyme-linked immunosorbent assay kits (Nanjing Jiancheng Bioengineering Institute, Nanjing, China). The content of malondialdehyde (MDA) and the activities of superoxide dismutase (SOD), glutathione peroxidase (GPX) and catalase (CAT) in the serum and jejunal mucosa samples as well as serum diamine oxidase (DAO) activity in the serum samples were determined using commercial kits (Nanjing Jiancheng Bioengineering Institute, Nanjing, China). All operations involved in these indicators were performed according to the manufacturer’s instructions by specific detection methods.

### 2.4. Intestinal Morphology

The paraformaldehyde-fixed jejunal samples were dehydrated, embedded, sliced and stained with Alcian blue/periodic acid-Schiff reagent (Nanjing Jiancheng Bioengineering Institute, Nanjing, China) as described previously [20]. Villus height (VH), crypt depth (CD), VH/CD and goblet cells were measured using the Leica DM3000 light microscope (Leica Microsystems, Wetzlar, Germany). At least 40 complete villus-crypt structures were measured in each sample.

### 2.5. Digestive Enzyme Measurement

The jejunal content was homogenized in precooled PBS by using a homogenizer, and the homogenate was centrifuged at 2500× *g* for 8 min at 4 °C to obtain the supernatant for the determination of the activities of amylase, lipase, trypsin and chymotrypsin. All enzyme activities and the supernatant’s protein concentration were measured using commercially available kits (Nanjing Jiancheng Bioengineering Institute, Nanjing, China) and specific detection methods according to the manufacturer’s protocols.

### 2.6. Real-time Polymerase Chain Reaction Analysis

Total RNA of each jejunal mucosa was extracted using TaKaRa’s TRIZOL reagent (Dalian, China) and then reverse-transcribed into cDNA by using the cDNA Synthesis kit (Yeasen, Shanghai, China) in accordance with the manufacturer’s instructions. Real-time PCR was performed on the ABI 7500 Real-Time polymerase chain reaction (PCR) System (Applied Biosystems, MA, USA) with SYBR Green qPCR Master Mix (Yeasen, Shanghai, China). The PCR reaction mixture contained 0.5 µL of the cDNA sample, 0.5 µL of each primer, 10 µL SYBR Green qPCR Master Mix and 9.5 µL of nuclease free water. The parameters for thermal cycling were set in accordance with our published study [21]. Primers of Nrf2, SOD, GPX, CAT, Keap-1, TLR4, NF-κB, IL-1β, IL-6, IL-8, IL-10, TNF-α, mucin-2, zonula occludens (ZO) -1, ZO-2, occludin, claudin-1, claudin-1 and glyceraldehyde-3 phosphate dehydrogenase (GAPDH) were obtained from other reports [19,22,23,24,25] and are listed showed in Table S2. All primers were commercially synthesized by Sangon Biotech Co., Ltd. (Shanghai, China). Target gene expression levels were analyzed according to the 2^−ΔΔCt^ method, and GAPDH was regarded as the internal gene.

### 2.7. Statistical Analysis

The general linear model was applied (Yij = µ + di + εij; Yij: the observation, µ: the general mean, di: the treatment effect, εij: the random error). Data were analyzed statistically using a one-way ANOVA followed by Duncan’s multiple range test by using SPSS 20.0 software (SPSS Inc., Chicago, IL, USA). Each replicate (*n* = 6) served as the experimental unit for growth performance, and the individual piglet (*n* = 6) served as the experimental unit for other indicators. Values are expressed as the means and the standard error of means (SEM). A *p* value of <0.05 indicated significant difference.

## 3. Results

### 3.1. Growth Performance, Diarrhea Rate and Immune Organ Indices

As presented in Table 1, no significant differences in the ADFI, F/G, thymus index and mesenteric lymph nodes index among groups were noted among the groups (*p* > 0.05). Compared with the CON group, the AGP, OAEO1 and OAEO2 groups had increased final BW and ADG values and decreased diarrhea rate (*p* < 0.05). Compared with the CON and AGP groups, the OAEO1 group had an increased spleen index (*p* < 0.05). The OAEO2 group showed a higher spleen index than did the CON group (*p* < 0.05).

### 3.2. Serum Immunity and Antioxidation Function

As displayed in Table 2, no significant differences in serum IL-1β, IL-6, IL-8 and IgM levels and CAT activity were noted among the groups (*p* > 0.05). Compared with the CON group, the AGP group had a higher IgA content and GPX activity; the OAEO1 group had higher IL-10, IgA and IgG contents and SOD and GPX activities and lower TNF-α and MDA contents; and the OAEO2 group had higher IL-10, IgA and IgG contents, and SOD activity, and a lower MDA content (*p* < 0.05). Compared with the AGP group, the OAEO1 group had a higher IL-10 content and a lower MDA content (*p* < 0.05).

### 3.3. Intestinal Morphology and Goblet Cell Numbers

As presented in Table 3, CD was not altered significantly among the groups (*p* > 0.05). No remarkable difference in jejunal morphology and goblet numbers was observed between the CON and AGP groups (*p* > 0.05). No remarkable changes were noted among the AGP, OAEO1 and OAEO2 groups (*p* > 0.05). Compared with the CON group, the OAEO1 group exhibited higher VH, VH/CD and goblet cell numbers. The OAEO2 group presented a higher VH/CD than the CON group (*p* < 0.05).

### 3.4. Digestive Enzyme Activity

As shown in Table 4, jejunal lipase and chymotrypsin activities were similar among the groups (*p* > 0.05). The AGP, OAEO1 or OAEO2 groups exhibited enhanced jejunal trypsin activity compared with the CON group (*p* < 0.05). The OAEO1 group demonstrated higher amylase activity than the CON group (*p* < 0.05).

### 3.5. Gut Barrier Function

As shown in Table 5, no significant differences in ZO-2 and claudin-2 mRNA levels among groups were noted among the groups (*p* > 0.05). Compared with the CON group, the AGP group exhibited lower serum DAO activity; the OAEO1 group exhibited higher ZO-1, occludin, claudin-1 and mucin-2 mRNA levels and a lower serum _D_-lactic acid content and DAO activity; and the OAEO2 group showed a higher ZO-1, occludin and mucin-2 mRNA levels and a lower serum _D_-lactic acid content and DAO activity (*p* < 0.05). Compared with the AGP group, the OAEO1 group had a higher occludin mRNA level and a lower _D_-lactic acid content, and the OAEO2 group showed higher ZO-1 and occludin mRNA levels (*p* < 0.05).

### 3.6. Jejunal Antioxidative Status and Nrf2 Signaling Pathway-Related Gene mRNA Levels

As presented in Table 6, no significant differences in SOD and CAT activities and keap-1, SOD and CAT mRNA levels among groups were observed among the groups (*p* > 0.05). No marked difference in jejunal antioxidative status and Nrf2 signaling pathway-related gene mRNA levels were observed between the CON and AGP groups (*p* > 0.05). Compared with the CON and AGP group, the OAEO1 and OAEO2 groups exhibited higher jejunal GPX activity and Nrf2 mRNA levels (*p* < 0.05). Compared with the CON group, the OAEO1 and OAEO2 groups had a lower MDA content and a higher GPX mRNA level (*p* < 0.05). In addition, compared with the AGP group, the OAEO1 group had a higher GPX mRNA level (*p* < 0.05).

### 3.7. Jejunal Immunity and TLR4/NFκB Signaling Pathway-Related Gene mRNA Levels

As presented in Table 7, no significant differences in IL-6 and IL-8 contents, and IL-1β, IL-6 and IL-8 mRNA levels were observed among the groups (*p* > 0.05). Compared with the CON group, the AGP group had a higher IL-10 content and mRNA level and a lower NF-κB mRNA level; the OAEO1 group had higher IL-10 content and mRNA level and lower IL-1β and TNF-α content and TLR4, NF-κB and TNF-α mRNA levels; and the OAEO2 group had a higher IL-10 mRNA level and lower IL-1β and TNF-α contents and NF-κB and TNF-α mRNA levels (*p* < 0.05). Compared with the AGP group, the OAEO1 group had a lower TNF-α content and TLR4 and TNF-α mRNA levels, and the OAEO2 group exhibited a lower TNF-α content (*p* < 0.05).

## 4. Discussion

Postweaning diarrhea is the main concern for early weaned piglets and leads to higher mortality and growth retardation. Traditionally, adding AGP to the feed is the most effective means to prevent diarrhea and enhance the growth performance of weaned pigs [6]; these effects were observed in this study. Because AGP have been widely banned, nutritional strategies must be developed to prevent diarrhea in weaned pigs [7]. In the past years, essential oils and organic acids have been widely used in animal production. One study found that essential oils treatment reduced the prevalence of postweaning diarrhea and improve the ADG of weaned piglets [26]. In addition, a lower diarrhea rate and higher growth performance were observed when weaned piglets were fed diets containing organic acids [27,28]. However, the use of the combination of different feed additives is a more promising nutritional strategy to replace AGP, because a single feed additive cannot completely achieve the effect of AGP [7]. The combination of organic acids and essential oils exerts a synergistic effect on the growth performance of weaned pigs, even exceeding the effect of AGP [29]. Similarly, Yang et al. [18] indicated that the blends of essential oils and organic acids considerably improved piglets’ ADG but had no remarkable effect on the diarrhea rate. However, Ma et al. [5] demonstrated that the mixture of essential oils and organic acids effectively alleviated diarrhea in piglets but exerted no significant effect on their growth performance. The results of this study indicate that dietary supplemented OAEO decreased the diarrhea rate and improved the ADG and final BW of the weaned piglets. These inconsistent results may be attributable to different feeding environments, types and concentrations of essential oil and organic acid, and even diet composition. The immune organ index, and serum IgG, IgA and IgM contents are crucial indicators of the immune function of animals [30,31,32]. Liu et al. [30] reported that a mixture of essential oils and organic acids raised the spleen index of broilers. Our results reveal that the spleen index and serum IgA and IgG levels were increased in OAEO groups compared with the CON group, indicating improvement in immune function; this might be a reason for the improved growth performance of weaned piglets by OAEO addition.

Intestinal morphological structures are paramount for intestinal digestion and absorption [1,26]. However, weaning stress results in the impairment of intestinal morphological structures [33]. Studies have reported that dietary essential oil supplementation improved the VH [26,29] and VH/CD of weaned piglets [26]. Long et al. [27] indicated that organic acids increased the VH and VH/CD of weaned piglets, while decreasing their CD. These findings suggest that the blend of organic acids and essential oils improves the morphological structure of the intestine. Previous studies on broilers [30] and weaned piglets [1] reported that the addition of organic acids and essential oils was feasible to improve intestinal morphology; this finding is consistent with those of this study. However, Xu et al. [29] and Ma et al. [5] indicated that organic acid and essential oil mixtures exerted no significant effect on weaned piglets’ gut morphology. The different types and composition ratios of organic acids and essential oils may be a major cause for the differences among results; therefore, additional studies are warranted. The deficiency of gastric acid and digestive enzyme activity is an important cause of diarrhea in piglets after weaning. Essential oils can promote the secretion of digestive enzymes, whereas acidifiers can increase the activities of digestive enzymes by reducing the gastrointestinal pH value [29,30,34]. A study on chickens reported that a combination of organic acids and essential oils considerably increased intestinal lipase, chymotrypsin and trypsin activities [30]. Another study on weaned piglets revealed that dietary organic acids and essential oils supplementation significantly enhanced trypsin and lipase activities in the pancreas and promoted the apparent total tract digestibility of dietary dry matter, organic matter and gross energy [5]. In our study, we observed that dietary organic acid and essential oil supplementation increased amylase and trypsin activities in the jejunal digesta. Therefore, OAEO improved the intestinal digestion and absorption capacity of weaned piglets; this, in turn, might have improved the growth performance and reduced diarrhea in the weaned piglets.

The intestinal epithelium barrier not only is critical for nutrient digestion and absorption but also acts a key defense against the invasion of antigens and pathogens in the intestinal lumen [22]. It is well documented that weaning stress severely impairs intestinal barrier function [35]. With the impairment of intestinal epithelium barrier, DAO as a cytoplasmic enzyme secreted by the intestinal epithelia and _D_-lactic acid as a product of intestinal bacteria enter the circulation of blood. Thus, they are the most commonly used blood markers to assess gut barrier function and permeability [22,36]. This study found that OAEO lowered serum DAO activity and _D_-lactic acid content, indicating a profitably decrease in the gut permeability of weaned piglets. The intestinal epithelium is covered with mucus, which is mainly secreted by goblet cells and protects the intestinal epithelium from pathogens and enterotoxin [36]. Ma et al. [5] revealed that the dietary supplementation of an organic acid and essential oil mixture up-regulated the mucin-2 (the main component of mucus) gene expression, which was demonstrated in this study. In addition, this study revealed that dietary supplemented OAEO significantly increased jejunal goblet cell numbers. Tight junctions between epithelial cells act as a selective permeable barrier and are mainly composed of ZO, occludin and claudin families; they play a crucial role in the intestinal barrier function [36]. The increase in intestinal permeability caused by the weaning stress of weaned piglets is implicated in the abnormal expression of intestinal tight junction [22,35]. Our study indicates that the addition of OAEO significantly up-regulated jejunal ZO-1, occludin and claudin-1 mRNA levels; this finding is similar to that reported by Ma et al. [5] who observed that a combination of organic acids and essential oils significantly increased ileal claudin-1 and occludin mRNA levels. These findings indicate that OAEO enhance the intestinal barrier function of weaned piglets partly by up-regulating tight junction-related genes and promoting mucus secretion, partly explaining why OAEO reduced the intestinal permeability of the weaned piglets in this study.

It is well documented that weaning stress may lead to an imbalance of the redox system and the accumulation of reactive oxygen species (ROS), ultimately resulting in oxidative injury (including in the gut) and inhibiting growth [37,38]. ROS is converted to hydrogen peroxide by SOD. Then, GPX and CAT convert hydrogen peroxide into oxygen and water [22]. Nrf2, a key transcription regulator, plays a key role in regulating antioxidant-related genes expression, including SOD, GPX and CAT. Therefore, the Nrf2 signaling pathway is essential for animals to cope with oxidative stress [21,39]. Ma et al. [5] found that dietary organic acid and essential oil supplementation increased SOD and GPX activities in serum, but had no significant effect on intestinal SOD and GPX activities and MDA (a lipid oxidation product) content. However, this study indicates that dietary OAEO supplementation not only increased serum SOD and GPX activities and reduced the MDA content in the serum and jejunal mucosa, but also increased jejunal GPX activity and mRNA level as well as Nrf2 mRNA level, indicating that OAEO can enhance the antioxidative capacity of weaned piglets and reduce weaning stress-induced oxidative damage by activating the Nrf2 signaling pathway, thereby possibly improving the intestinal barrier function of the weaned piglets.

An abnormal inflammatory response is one of the adverse outcomes of weaning stress [38], which is detrimental to the growth performance and gut function of weaned piglets [22,40] because proinflammatory cytokines can regulate gut permeability and intestinal tight junctions’ expression [41]. TLR4 is a typical member of the pattern-recognition receptors family, and can be activated by endotoxin. TLR4 causes systemic inflammation by activating NF-κB, which triggers the expression of a string of inflammation-related genes, including IL-1β, IL-2, IL-6, IL-8 and TNF-α [42]. IL-10 is a crucial anti-inflammatory cytokine that can inhibit NF-κB activity and modulate the inflammatory process [5,43]. A previous study found that the dietary supplementation of a combination of essential oils and organic acids increased the serum IL-10 level in weaned piglets [5], which was confirmed by this study. The findings indicate that a blend of essential oils and organic acids exerts an anti-inflammatory effect. Moreover, we observed that OAEO reduced IL-1β and TNF-α contents and TLR4 and NF-κB mRNA levels and increased the IL-10 content and mRNA level in the jejunum mucosa of the weaned piglets. These positive findings suggest that OAEO increase the anti-inflammatory capacity and alleviate weaning stress-induced inflammatory responses, probably by suppressing the activation of the TLR4/NF-κB signaling pathway, which might be another reason why OAEO enhance the intestinal barrier function of weaned piglets as found above.

## 5. Conclusions

Dietary OAEO supplementation decreased the incidence of diarrhea and increased the growth performance, immuno-antioxidant function and intestinal health, which were reflected by improved intestinal morphological structures, elevated digestive enzyme activities and enhanced barrier function. The mechanisms underlying the improved intestinal health in response to OAEO might be partly linked to the activation of the intestinal Nrf2 signaling pathway, which improves the intestinal antioxidative capacity, and the suppression of the NF-κB pathway, which inhibit excessive inflammatory responses. Therefore, OAEO can be added to weaned piglets’ diet as alternative to AGP. However, further investigation is needed to elucidate the exact mechanism of action.

## Figures and Tables

**Table 1:** Effect of OAEO on growth performance, diarrhea rate and immune organ indices of weaned piglets.

Items	CON	AGP	OAEO1	OAEO2	SEM	*p*-Value
Initial BW, kg	6.97	6.98	6.96	6.93	0.016	0.741
Final BW, kg	12.48 ^b^	13.22 ^a^	13.43 ^a^	13.29 ^a^	0.122	0.015
ADFI, g	383.1	420.4	436.3	424.8	7.907	0.085
ADG, g	261.1 ^b^	297.5 ^a^	308.2 ^a^	303.0 ^a^	5.920	0.014
F/G	1.46	1.41	1.42	1.40	0.009	0.113
Diarrhea rate, %	26.71 ^a^	13.15 ^b^	14.65 ^b^	12.52 ^b^	1.316	<0.001
Spleen index, g/kg	1.18 ^c^	1.27 ^bc^	1.91 ^a^	1.66 ^ab^	0.092	0.007
Thymus index, g/kg	1.22	1.16	1.33	1.53	0.061	0.168
Mesenteric lymph nodes index, g/kg	1.49	1.55	1.44	1.35	0.077	0.837

Means (*n* = 6) within a row with different superscript letters differ significantly (*p* < 0.05). CON: basal diet group; AGP: basal diet + 10 mg/kg bacitracin zinc + 20 mg/kg colistin sulphate; OAEO1: basal diet + 1 g/kg encapsulated organic acid and essential oil mixture; OAEO2: basal diet + 2 g/kg encapsulated organic acid and essential oil mixture. SEM, standard error of the mean; BW, body weight; ADFI, average daily feed intake; F/G, ratio of feed intake to body weight gain; ADG, average daily gain.

**Table 2:** Effect of OAEO on serum immunity and antioxidation function of weaned piglets.

Items	CON	AGP	OAEO1	OAEO2	SEM	*p*-Value
IL-1β, ng/L	93.15	96.19	89.73	92.84	1.634	0.609
IL-6, ng/L	108.3	105.9	107.6	103.1	1.803	0.773
IL-8, ng/L	41.95	43.50	45.16	42.35	0.943	0.655
IL-10, ng/L	12.16 ^c^	13.98 ^bc^	18.18 ^a^	16.35 ^ab^	0.675	0.003
TNF-α, ng/L	66.37 ^a^	58.20 ^b^	56.26 ^b^	60.70 ^ab^	1.287	0.021
IgA, g/L	0.59 ^b^	0.87 ^a^	0.90 ^a^	0.82 ^a^	0.034	0.002
IgG, g/L	3.21 ^b^	4.08 ^ab^	4.82 ^a^	4.64 ^a^	0.216	0.014
IgM, g/L	1.18	1.17	1.33	1.31	0.041	0.363
SOD, U/mL	26.05 ^b^	29.99 ^ab^	32.51 ^a^	33.31 ^a^	1.043	0.048
CAT, U/mL	51.99	50.70	56.04	53.65	1.353	0.564
GPX, U/mL	365.9 ^b^	427.8 ^a^	410.0 ^a^	391.2 ^ab^	7.531	0.014
MDA, nmol/mL	1.89 ^a^	1.76 ^ab^	1.33 ^c^	1.46 ^bc^	0.073	0.013

Means (*n* = 6) within a row with different superscript letters differ significantly (*p* < 0.05). CON: basal diet group; AGP: basal diet + 10 mg/kg bacitracin zinc + 20 mg/kg colistin sulphate; OAEO1: basal diet + 1 g/kg encapsulated organic acid and essential oil mixture; OAEO2: basal diet + 2 g/kg encapsulated organic acid and essential oil mixture. IL-1β: interleukin-1β; IL-6: interleukin-6; IL-8: interleukin-8; IL-10: interleukin-10; TNF-α: tumor necrosis factor-α; IgA: immunoglobulin A; IgG: immunoglobulin G; IgM: immunoglobulin M; SOD: superoxide dismutase; CAT: catalase; GPX: glutathione peroxidase; MDA: malondialdehyde.

**Table 3:** Effect of OAEO on intestinal morphology and goblet cell numbers of piglets.

Items	CON	AGP	OAEO1	OAEO2	SEM	*p*-Value
Villus height, μm	292.4 ^b^	310.1 ^ab^	338.3 ^a^	320.8 ^ab^	5.520	0.016
Crypt depth, μm	158.5	151.4	147.3	143.5	4.110	0.639
Villus height/crypt depth	1.87 ^b^	2.07 ^ab^	2.33 ^a^	2.26 ^a^	0.061	0.020
Goblet cell numbers	16.51 ^b^	18.30 ^ab^	22.28 ^a^	19.94 ^ab^	0.761	0.024

Means (*n* = 6) within a row with different superscript letters differ significantly (*p* < 0.05). CON: basal diet group; AGP: basal diet + 10 mg/kg bacitracin zinc + 20 mg/kg colistin sulphate; OAEO1: basal diet + 1 g/kg encapsulated organic acid and essential oil mixture; OAEO2: basal diet + 2 g/kg encapsulated organic acid and essential oil mixture.

**Table 4:** Effect of OAEO on intestinal digestive enzyme activities of piglets.

Items	CON	AGP	OAEO1	OAEO2	SEM	*p*-Value
Amylase, U/g protein	44.55 ^b^	49.22 ^ab^	60.59 ^a^	54.83 ^ab^	2.256	0.046
Lipase, U/g protein	18.38	21.33	19.23	21.53	0.946	0.594
Trypsin, U/g protein	12.02 ^b^	20.35 ^a^	23.94 ^a^	19.35 ^a^	1.224	0.001
Chymotrypsin, U/g protein	24.69	22.03	25.84	27.16	1.269	0.557

Means (*n* = 6) within a row with different superscript letters differ significantly (*p* < 0.05). CON: basal diet group; AGP: basal diet + 10 mg/kg bacitracin zinc + 20 mg/kg colistin sulphate; OAEO1: basal diet + 1 g/kg encapsulated organic acid and essential oil mixture; OAEO2: basal diet + 2 g/kg encapsulated organic acid and essential oil mixture.

**Table 5:** Effect of OAEO on gut barrier integrity-related genes mRNA levels, and serum _D_-lactic acid content and diamine oxidase activity of piglets.

Items	CON	AGP	OAEO1	OAEO2	SEM	*p*-Value
_D_-Lactic acid, µg/mL	24.48 ^a^	22.32 ^ab^	17.15 ^c^	18.97 ^bc^	0.830	0.002
Diamine oxidase, U/L	12.07 ^a^	9.92 ^b^	8.71 ^b^	9.33 ^b^	0.418	0.016
ZO-1	1.00 ^c^	1.16 ^bc^	1.61 ^ab^	1.71 ^a^	0.100	0.02
ZO-2	1.00	0.93	1.25	1.05	0.062	0.318
Occludin	1.00 ^b^	1.11 ^b^	1.78 ^a^	1.82 ^a^	0.108	0.002
Claudin-1	1.00 ^b^	1.19 ^ab^	1.73 ^a^	1.32 ^ab^	0.102	0.041
Claudin-2	1.00	0.85	1.25	1.05	0.067	0.214
Mucin-2	1.00 ^b^	1.29 ^ab^	1.73 ^a^	1.77 ^a^	0.107	0.018

Means (*n* = 6) within a row with different superscript letters differ significantly (*p* < 0.05). CON: basal diet group; AGP: basal diet + 10 mg/kg bacitracin zinc + 20 mg/kg colistin sulphate; OAEO1: basal diet + 1 g/kg encapsulated organic acid and essential oil mixture; OAEO2: basal diet + 2 g/kg encapsulated organic acid and essential oil mixture. ZO-1: zonula occludens-1; ZO-2: zonula occludens-2.

**Table 6:** Effect of OAEO on intestinal antioxidant status and Nrf2 signaling pathway-related gene mRNA levels of weaned piglets.

Items	CON	AGP	OAEO1	OAEO2	SEM	*p*-Value
SOD, U/mg protein	56.13	58.85	67.13	59.35	1.747	0.097
CAT, U/mg protein	4.82	4.71	5.34	4.77	0.172	0.566
GPX, U/mg protein	14.73 ^b^	14.67 ^b^	20.03 ^a^	20.51 ^a^	0.778	0.001
MDA, nmol/mg protein	1.96 ^a^	1.78 ^ab^	1.40 ^b^	1.47 ^b^	0.076	0.018
Nrf2	1.00 ^b^	0.96 ^b^	1.72 ^a^	1.57 ^a^	0.112	0.015
Keap-1	1.00	0.86	0.83	0.80	0.084	0.871
SOD	1.00	1.16	1.58	1.09	0.085	0.065
GPX	1.00 ^c^	1.12 ^bc^	1.72 ^a^	1.59 ^ab^	0.108	0.033
CAT	1.00	0.82	0.91	0.89	0.072	0.863

Means (*n* = 6) within a row with different superscript letters differ significantly (*p* < 0.05). CON: basal diet group; AGP: basal diet + 10 mg/kg bacitracin zinc + 20 mg/kg colistin sulphate; OAEO1: basal diet + 1 g/kg encapsulated organic acid and essential oil mixture; OAEO2: basal diet + 2 g/kg encapsulated organic acid and essential oil mixture. SOD: superoxide dismutase; CAT: catalase; GPX: glutathione peroxidase; MDA: malondialdehyde; Nrf2: nuclear factor erythroid 2-related factor 2.

**Table 7:** Effect of OAEO on intestinal cytokine contents and TLR-4/NF-κB signaling pathway-related gene mRNA levels of weaned piglets.

Items	CON	AGP	OAEO1	OAEO2	SEM	*p*-Value
IL-1β, ng/g protein	38.12 ^a^	36.47 ^ab^	30.92 ^b^	30.67 ^b^	1.223	0.048
IL-6, ng/g protein	60.67	57.29	55.53	56.02	1.608	0.697
IL-8, ng/g protein	17.06	17.91	19.38	18.44	0.663	0.685
IL-10, ng/g protein	8.40 ^b^	11.80 ^a^	12.67 ^a^	11.30 ^ab^	0.554	0.037
TNF-α, ng/g protein	30.30 ^a^	28.89 ^a^	23.22 ^b^	22.35 ^b^	0.986	0.004
TLR4	1.00 ^a^	1.02 ^a^	0.63 ^b^	0.67 ^ab^	0.066	0.045
NF-κB	1.00 ^a^	0.66 ^b^	0.70 ^b^	0.69 ^b^	0.051	0.048
IL-1β	1.00	1.10	0.80	0.61	0.071	0.061
IL-6	1.00	1.02	0.91	0.97	0.082	0.977
IL-8	1.00	1.00	1.14	0.91	0.079	0.799
IL-10	1.00 ^b^	1.49 ^a^	1.61 ^a^	1.68 ^a^	0.082	0.005
TNF-α	1.00 ^a^	0.97 ^ab^	0.61 ^c^	0.67 ^bc^	0.059	0.025

Means (*n* = 6) within a row with different superscript letters differ significantly (*p* < 0.05). CON: basal diet group; AGP: basal diet + 10 mg/kg bacitracin zinc + 20 mg/kg colistin sulphate; OAEO1: basal diet + 1 g/kg encapsulated organic acid and essential oil mixture; OAEO2: basal diet + 2 g/kg encapsulated organic acid and essential oil mixture. TLR4: Toll-like receptor 4; NF-κB: nuclear factor-κB; IL-1β: interleukin-1β; IL-6: interleukin-6; IL-8: interleukin-8; IL-10: interleukin-10; TNF-α: tumor necrosis factor-α.

## Data Availability

The data presented in this study are available upon request from the corresponding author.

## Supplementary Materials

The following supporting information can be downloaded at: https://www.mdpi.com/article/10.3390/ani12182426/s1, Table S1: Ingredient composition and nutrient content of the basal diet; Table S2: Primer sequences for target and housekeeping genes.
