# Supplementary material for: An Encapsulated Organic Acid and Essential Oil Mixture Improves the Intestinal Health of Weaned Piglets by Altering Intestinal Inflammation and Antioxidative Capacity
Source: Animals (Basel). 2022 Sep 15;12(18):2426. doi: 10.3390/ani12182426 (PMC9495186; doi:10.3390/ani12182426)
Supplement: Supplementary file 1 [file animals-12-02426-s001.zip › animals-1898224-Supplementary.pdf]

Table S1. Ingredient composition and nutrient content of basal diet.

Ingredient, %		Calculated Nutrient Content	
Corn	31.70	Digestible energy, MJ/kg	14.78
Extruded corn	32.60	Crude protein, %	19.49
Extruded soybean	15.00	Calcium, %	0.71
Soybean protein concentrate	11.00	Available phosphorus, %	0.34
Fish meal	3.00	SID[2] lysine, %	1.21
Sucrose	2.50	SID[2] threonine, %	0.66
Glucose	1.00	SID[2] methionine + cysteine, %	0.61
Limestone	0.90		
Dicalcium phosphate	0.60		
Soybean oil	0.50		
L-Lysine HCl	0.30		
Salt	0.30		
DL-Methionine	0.05		
Choline chloride	0.10		
Non-antibiotic premix[1]	0.45		

[1] Non-antibiotic premix included per kilogram of diet: 16000 IU vitamin A, 3000 IU vitamin D_3, 38.5 mg vitamin E, 3.5 mg vitamin K, 3.5 mg vitamin B_1, 10.0 mg vitamin B_2, 5.5 mg vitamin B_6, 0.10 mg vitamin B_{12}, 3.0 mg folic acid, 35 mg nicotinamide, 0.25 mg biotin, 30 mg pantothenic acid, 100 mg Fe ($FeSO_4 \cdot H_2O$), 6 mg Cu ($CuSO_4 \cdot 5H_2O$), 4 mg Mn ($MnSO_4 \cdot H_2O$), 100 mg Zn ($ZnSO_4 \cdot H_2O$), 0.14 mg I (KI), 0.35 mg Se (Na_2SeO_3).

[2] Standardized ileal digestible.

Table S2. Primer Sequences for target and housekeeping genes.

Genes	Primer sequences (5'-3')	Product length (bp)
Nrf2	Forward: CACCACCTCAGGGTAATA	125
	Reverse: GCGGCTTGAATGTTTGTC	
Keap-1	Forward: ACGACGTGGAGACAGAAACGT	56
	Reverse: GCTTCGCCGATGCTTCA	
SOD	Forward: ATTCTGTGATCGCCCTCT	100
	Reverse: CTTTCTTCATTTCCACCTCT	
GPX	Forward: TTGCCAAGTCCTTCTACGA	188
	Reverse: GAAGCCAAGAACCACCAG	
CAT	Forward: CGAAGGCGAAGGTGTT	109
	Reverse: CCACGAGGGTCACGAA	
TLR4	Forward: TCAGTTCTCACCTTCCTCCTG	166
	Reverse: GTTCATTCCTCACCCAGTCTTC	
NF-κB	Forward: AGTACCCTGAGGCTATAACTCGC	133
	Reverse: TCCGCAATGGAGGAGAAGTC	
IL-1β	Forward: CCTGGACCTTGGTTCTCT	123
	Reverse: GGATTCTTCATCGGCTTCT	
IL-6	Forward: GGCAAAAGGGAAAGAATCCAG	87
	Reverse: CGTTCTGTGACTGCAGCTTATCC	
IL-8	Forward: CACCTGTCTGTCCACGTTGT	126
	Reverse: AGAGGTCTGCCTGGACCCCA	
IL-10	Forward: GGGCTATTTGTCCTGACTGC	105
	Reverse: GGGCTCCCTAGTTTCTCTTCC	
TNF-α	Forward: ACAGGCCAGCTCCCTCTTAT	102
	Reverse: CCTCGCCCTCCTGAATAAAT	
Mucin-2	Forward: AGACGGGCGGAGACTTTGAATC	102
	Reverse: CTTGGATGGGAACGCTGGGATA	
ZO-1	Forward: CAGCCCCCGTACATGGAGA	114
	Reverse: GCGCAGACGGTGTTCATAGTT	
ZO-2	Forward: ATTCGGACCCATAGCAGACATAG	90
	Reverse: GCGTCTCTTGGTTCTGTTTTAGC	
Occludin	Forward: CTACTCGTCCAACGGGAAAG	158
	Reverse: ACGCCTCCAAGTTACCACTG	
Claudin-1	Forward: GCCACAGCAAGGTATGGTAAC	140
	Reverse: AGTAGGGCACCTCCCAGAAG	
Claudin-2	Forward: GCATCATTTCCTCCCTGTT	156
	Reverse: TCTTGGCTTTGGGTGGTT	
GAPDH	Forward: TGAAGGTCGGAGTGAACGGAT	114
	Reverse: CACTTTGCCAGAGTTAAAAGCA	

Nrf2: nuclear factor erythroid 2-related factor 2; SOD: superoxide dismutase; GPX: glutathione peroxidase; CAT: catalase; TLR4: Toll-like receptor 4; NF-κB: nuclear factor-κB; IL-1β: interleukin-1β; IL-6: interleukin-6; IL-8: interleukin-8; IL-10: interleukin-10; TNF-α: tumor necrosis factor-α; ZO-1: zonula occludens-1; ZO-2: zonula occludens-2; GAPDH: glyceraldehyde-3 phosphate dehydrogenase.
